# Novel Multi-View RGB Sensor for Continuous Motion Analysis in Kinetic Chain Exercises: A Pilot Study for Simultaneous Validity and Intra-Test Reliability

**DOI:** 10.3390/s23249635

**Published:** 2023-12-05

**Authors:** Junghoon Ahn, Hongtaek Choi, Heehwa Lee, Jinyoung Lee, Hyeong-Dong Kim

**Affiliations:** 1 Department of Health Science, Graduate School, Korea University, Seoul 02841, Republic of Korea; physioahn@korea.ac.kr (J.A.); dr_choi@korea.ac.kr (H.C.); 2 Department of Sports Convergence, Sangmyung University, Cheonan 31066, Republic of Korea; leehh@smu.ac.kr; 3 Department of Green Chemical Engineering, Sangmyung University, Cheonan 31066, Republic of Korea

**Keywords:** 4DEYE^®^, VICON^®^, human kinetic chain, side dip, Y-balance

## Abstract

As the number of musculoskeletal disorders caused by smartphone usage, sedentary lifestyles, and active sports activities increases, there is a growing demand for precise and accurate measurement and evaluation of issues such as incorrect compensation patterns, asymmetrical posture, and limited joint operation range. Urgent development of new inspection equipment is necessary to address issues such as convenience, economic feasibility, and post-processing difficulties. Using 4DEYE^®^, a new multi-view red, green, and blue (RGB) sensor-based motion analysis equipment, and the VICON^®^ ratio, which are infrared-based markers, we conducted a comparative analysis of the simultaneous validity of the joint angle (trajectory) and reliability. In this study, five healthy participants who could perform movements were selected for the pilot study and two movements (Y-balance and side dip) were analyzed. In addition, the ICC (Intraclass Correlation Coefficient) was analyzed using the SPSS (Statistical Package for the Social Sciences) V.18 while the number of data frames of each equipment was equalized using the MATLAB program. The results revealed that side dips, which are open kinetic chain exercises (intraclass correlation coefficient ICC(2.1), 0.895–0.996), showed very high concordance with the Y-balance test, a closed kinetic chain exercise (ICC(2.1), 0.678–0.990). The joint measurement results were similar regardless of the movement in the open or closed kinetic chain exercise, confirming the high reliability of the newly developed multiview RGB sensor. This is of great significance because we obtained important and fundamental results that can be used in various patterns of exercise movements in the future.

## 1. Introduction

Accurate analysis of dynamic motion in fields such as automobile navigation, robot control, and wearable devices is one of the key factors in increasing technological progress and applicability, while dynamic motion evaluation is one of the important ways to precisely measure and evaluate human motion [1]. This need is mainly related to Inertial Measurement Units (IMUs). IMUs are devices composed of various sensors such as accelerometers, gyroscopes, and magnetometers, which are used to accurately measure the motion of objects. IMUs are applied to acceleration, rotation, and location tracking of vehicles in car navigation, whereas robot control utilizes robot motion and posture control. It is also utilized to monitor user movements and activities in wearable devices [2]. However, IMUs often have their limitations such as the accuracy and the sensor drift. Therefore, more sophisticated algorithms are needed with the support of advanced sensor technology to perform dynamic motion evaluation more precisely. As the demand for precise and accurate methods for evaluating human movement has continued to increase over the past decade, the importance of evaluating postural or movement damage to address musculoskeletal dysfunction has recently been recognized [3]. Using the latest technologies and equipment, various studies have analyzed compensation and abnormal posture, which involve movements outside the body’s established standard range [4,5,6]. Analyzing damaged human movements and asymmetric postures can quickly identify injury risk factors, reducing patient recovery time and enabling more effective treatment plans [7,8]. Most published studies involving human kinematics have used three-dimensional (3D) motion analysis systems, which are expensive, time consuming, and generally not widely available in the clinical field [9]. VICON^®^, a typical 3D motion analysis system, is the most commonly used standardized approach in motion analysis. Nevertheless, it can be time consuming because of the expensive device, limited inspection environment, various limitations involving the use of sensors and markers [10], and expertise for equipment use. With the development of related technologies, low-cost and efficient solutions have emerged for evaluating the human body. Two-dimensional (2D) red, green, and blue (RGB) sensors have become affordable alternatives to 3D systems [11,12]. The 2D RGB sensor is cheap and easy to carry; therefore, it is useful for simple motion analysis or measurement. However, the reliability of the measurement value is significantly reduced for precise motion analysis.

To address this issue, recent studies have explored a system that accurately measures operations in three dimensions using three or more inexpensive RGB sensors, eliminating the need for markers. In the case of a single-view RGB sensor, 4DEYE^®^ has issues with deep ambiguity and occlusion according to the timing. Therefore, considering the deep learning network package of motion recognition through OpenPose, key points of each joint of the object were labeled as continuous images based on multiple views and defined as a standard dataset. In addition, research is underway to develop a 3D skeleton model acquisition technology that includes the 3D joint angle and location information of major joints in the body by training a deep learning-based body joint estimation model through image collection using three or more RGB sensors. Furthermore, the correlation between the main joint positions of the body that appear during movement can be identified, patterned into a database, and used to generate positions and angles of major joints in frame units from continuous movements, such as walking. This information, along with a motion unit recognition algorithm, is used to compare the correlation between each joint in a single movement. Additionally, it has a quantitative evaluation technology for user movements that stores user motion data generated through body joint estimation, analyzes continuous posture over time, defines a function for an appropriate distance, and compares it with existing stored reference movements [13]. 

As this study combines clinical and engineering research, it is important to understand the anatomical plane and human kinetic chain. Anatomy defines three basic planes that describe the axes on which human movement occurs. The first is called the sagittal plane, which is perpendicular to the ground and divides the body into the left and right sides. Flexion and extension typically occur in this plane. Second, the front plane was perpendicular to the ground, and the body was divided into the dorsal (back) and ventral (front) parts. Abduction and adoption typically occur in this plane. Third, the transverse plane divides the body into the cranial (head) and caudal (tail) planes. It is parallel to the ground, separates the upper and lower parts, and is further divided into the superior and inner parts. Rotation typically occurs in this plane [6,7,14,15,16]. To explain human movement, the kinetic chain is divided into an open kinetic chain (OKC) and a closed kinetic chain (CKC). It is located in the distal part (e.g., hand) of the OKC and is free without being fixed to any object [17]. In the CKC, the distal part of the body is fixed. These two types of motion chain exercises have several advantages. The biggest advantage of OKC exercise is that muscles can be individually exercised, while in CKC exercise, the participants can simultaneously conduct resistance training on the distal and proximal parts of the body and stimulate the proximal system to initiate and control muscle activation patterns [18,19]. 

The pilot study’s aim is that the movement angle of the hip extension motion was measured, formed through a combination of the CKC and sagittal plane movements using 4DEYE^®^, a new markerless multi-view image-based motion analysis system, and the trunk side-bending motion formed by movements, such as the OKC and front plane. The number of frames of 4DEYE^®^ is lower than that of VICON^®^, and simple low-speed and single-plane operations are suitable rather than complex high-speed operations, so an operation experiment was conducted accordingly. The research hypothesis is that the measurement would establish the similarity validity of the simultaneous and angular trajectories of the VICON^®^ motion capture system with a marker and 4DEYE^®^, a novel marker multi-view image-based motion analysis system. 

## 2. Materials and Methods

### 2.1. Participants

Five healthy young people (age, 25.4 ± 2.0 years; body height, 174.4 ± 5.0 cm; body mass, 68.9 ± 6.8 kg) who could accurately and continuously perform experimental tasks, such as knee bending movements and deep squat movements when sitting and standing, voluntarily participated in the pilot study. Those with a history of neurological, musculoskeletal, or cognitive disorders were excluded. Prior to study initiation, the participants were informed concerning its purpose and procedure and submitted a signed consent form. The experimental protocol followed the Declaration of Helsinki [20] and was approved by the Institutional Review Board of a Korea University (KUIRB-2022-0260-01) prior to implementation. Five RGB cameras (4DEYE^®^, SYM Healthcare Inc., Seoul, Republic of Korea) were used for the multi-view image-based motion analysis. 

### 2.2. Measurements

**Figure 1:**
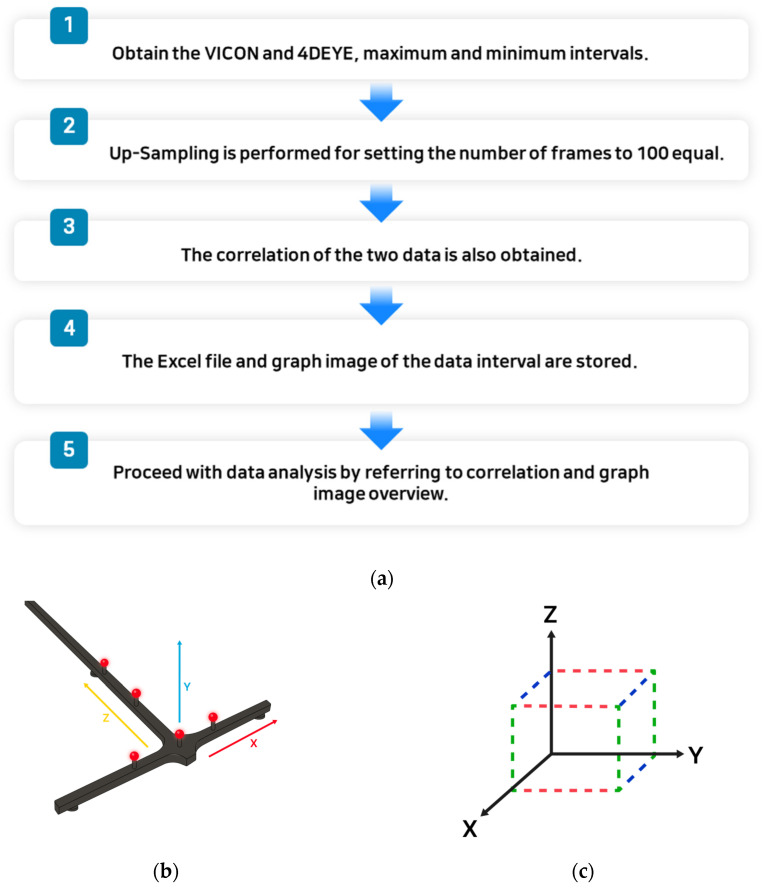
(**a**) Analysis process; (**b**) matching VICON^®^; (**c**) matching 4DEYE^®^.

Accurately determining the timing of the operation using the 4DEYE^®^ and VICON^®^ systems was challenging. Therefore, the maximum and minimum sections of each data point were simultaneously measured and compared. After selecting one representative indicator for 4DEYE^®^ and VICON^®^, the maximum and minimum sections of the indicator were applied to all indicators. The measurements were performed as shown in Figure 1a.

The measurement methods using the 4DEYE^®^ and VICON^®^ systems were different; therefore, there was a slight difference in the angle values. In addition, artificial errors may have occurred since the start and end times of the 4DEYE^®^ and VICON^®^ devices were manually operated by the researcher, not automated. VICON^®^ proceeded with the processed data after performing; in that case, the number of data frames was unified to 100. However, the data that were not processed after the performance showed a relatively large angle difference from 4DEYE^®^. When matching VICON^®^ (Figure 1b) and 4DEYE^®^ (Figure 1a,b), it was proceeded by referring to these images.

### 2.3. VICON^®^ Motion Capture System

The VICON^®^ Motion Capture System (MXT series, Oxford Metrics, Ltd., Oxford, UK) includes 8 infrared cameras and has dedicated hardware that uses them to locate coordinates of location points. The VICON^®^ system also includes a counter-reflection marker depending on the infrared signal from the infrared strobe of each camera. The torso and lower limb landmarks consist of four attached markers (14 mm in diameter), as well as the seventh cervical spine (C7), eighth thoracic spine (T8), and xiphoid processes of the jugular and sternum. In addition, two cross-shaped clusters consisting of four markers were attached to the thigh and shank, and one axis of the cross was measured due to alignment with the femur or tibia axis. In addition, each camera analyzed the 3D position of all markers in the 100 Hz range, and the joint angle was calculated in a similar way to measurements based on a multi-view motion capture system. The body coordinates, femoral axis, and tibia axis vectors were set using the position points of each marker, and the body coordinates were set according to Wu et al. [21]. Cross-shaped clusters were used for femur and tibia axis vectors, while MATLAB R2018A (The Mathworks, Inc., Natick, MA, USA) was used for joint angle measurements.

### 2.4. Multi-View Image-Based Motion Analysis System (4DEYE^®^)

Using a multi-view image collection system (4DEYE^®^, SYM Healthcare Inc., Seoul, Republic of Korea) consisting of four RGB cameras, patients’ poses were photographed at 12 Hz in four different directions. Therefore, images of movements from various directions were collected, and the angles of the hip and knee joints were analyzed using a user-defined analysis program developed based on OpenCV [22] and OpenPose [23]. Specifically, the OpenPose software acquired five images simultaneously using five cameras to estimate the 2D positions of all seven physical key points, including the neck, left shoulder, right shoulder, right hip, knee, and ankle. Then, OpenCV software reconstructed the 3D position of each key point from four different 2D positions of the key point based on information on the relative position and orientation of the camera. The bending and stretching of the hip joint could be explained by the angle of the thigh axis for the entire body, and the bending or stretching of the knee could be explained by the angle between the thigh and the tibial axis. The body coordinates were obtained as follows: The Z-axis of the body was obtained as a vector pointing from the joint to the neck, and the X-axis was defined as a vector perpendicular to the plane consisting of the left shoulder, right shoulder, and used joint. The addition and Y axis were defined as vectors orthogonal to the Z and X axes, and the femur and tibia axis vectors were defined as vectors pointing to the knee at the right hip and ankle at the knee, respectively. The hip flexion angle was calculated as the angle between the negative Z-axis of the torso coordinate and the thigh axis vector to quantify the hip flexion in a 3D space regardless of the plane of hip flexion. As the leg was raised, the hip flexion angle increased from 0° (i.e., anatomically neutral posture) to 180°. Finally, the calculated joint angle was calibrated to match the data length with data collected at 100 Hz using the VICON^®^ motion capture system.

### 2.5. Procedure

**Figure 2:**
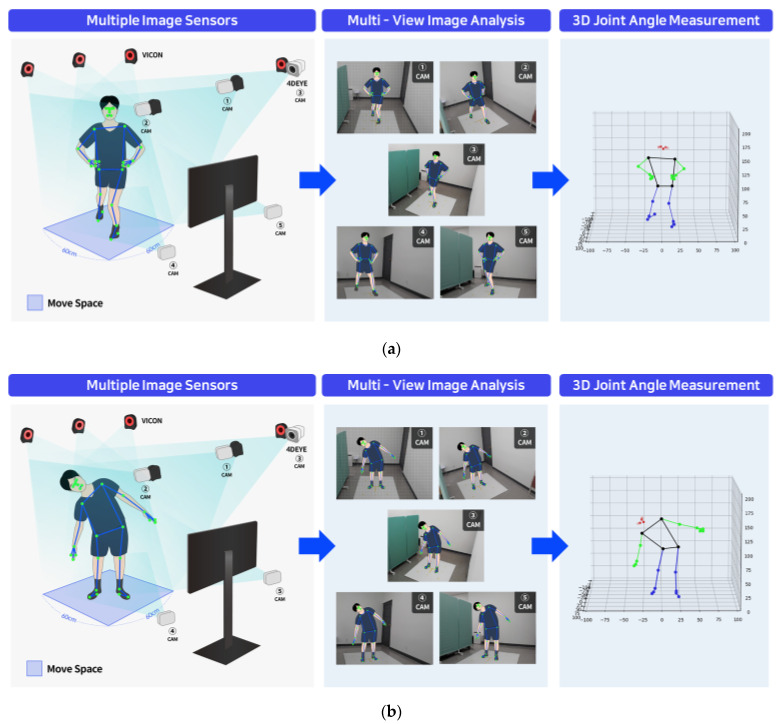
Multi-view image-based 4DEYE^®^ analysis system. (**a**) Y-balance (posterior-lateral). (**b**) Side dip (standing).

In this experiment, the participants performed high-level connection motions (i.e., side dip and Y-balance test [YBT]) using multi-joints [24,25]. The experimenter explained and demonstrated the motions to the participant, who then practiced these movements before the experiment. Side dip is an OKC exercise, with both shoulders lightly abducted by approximately 30°. The trunk performed spatial flexion in a neutral position with minimal flexion, extension, and rotation, and the participants were instructed to perform side-bending until the opposite oblique muscle felt tight. The YBT is a CKC exercise where both hands are fixed to the waist, support to the lower extremities is provided by hip flexion and knee flexion, and the moving lower extremities are maintained at 0° of knee extension while reaching out is performed in the posterolateral direction (diagonal 30°) [26,27]. The motion was repeated five times, and the two capture systems were simultaneously activated when the participant started and completed the motion simultaneously while maintaining the final posture. The break interval was 3–5 min. A researcher with a physical therapist license conducted the inspection and management directly to secure the participant’s performance ability and to maintain the accuracy and best condition of the motion. Joint angle data for hip extension and trunk spatial flexion were collected and processed (Figure 2). 

### 2.6. Statistical Analysis

The results are presented as means and standard deviations (SDs). Simultaneous and angle-limited validity (intraclass correlation coefficient; ICC3, k) of the new multi-view image-based motion analysis system (marker-less) developed in this study was compared with that of the VICON^®^ motion capture system (including markers). ICC analysis used ICCs and 95% confidence intervals (CIs), and ICC analysis was used to evaluate the in-screen reliability (ICC3, 1) of each motion analysis system. ICC values were interpreted as ICC < 0.50 (defect), 0.50–0.75 (defect), 0.76–0.90 (good), and 0.90 (high). We also calculated the coefficient of variation (CV), standard error of measurement (SEM), and minimum detectable change (MDC) to determine the absolute reliability [27]. In addition, the CV for the method error was calculated as follows:

$$\mathrm{CV}=100\times (2\times (\mathrm{SDD}/\sqrt{2}))/(X1+X2),$$

where SD is the mean of each of the two measures [28].

SEM was calculated as follows:

$$\mathrm{SEM}=\mathrm{SD}\times \sqrt{(1-\mathrm{ICC})}.$$

Microsoft Excel 2019 (Microsoft Inc., Redmond, WA, USA) was used to provide measurements of variability. Finally, MDC presents the smallest statistical estimate of variation, providing confidence that is not a result of target volatility or measurement errors, calculated as a pole variable: MDC = z score (95% CI) × SEM × $\sqrt{2}$ [29]. The significance level was set at *p* < 0.05. All statistical analyses were performed using SPSS for Windows (version 18.0; SPSS Inc., Chicago, IL, USA) and Microsoft Excel 2019 (Microsoft Inc.).

## 3. Results 

### 3.1. Analysis of Side Dip

**Figure 3:**
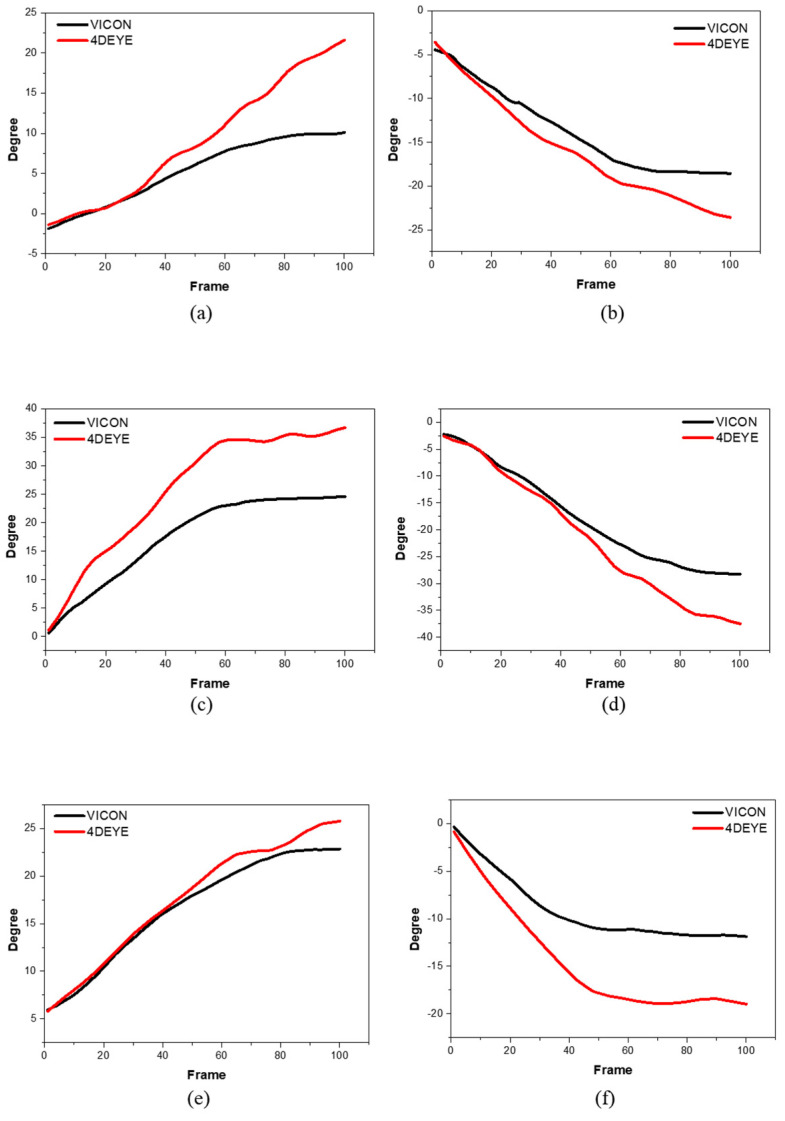

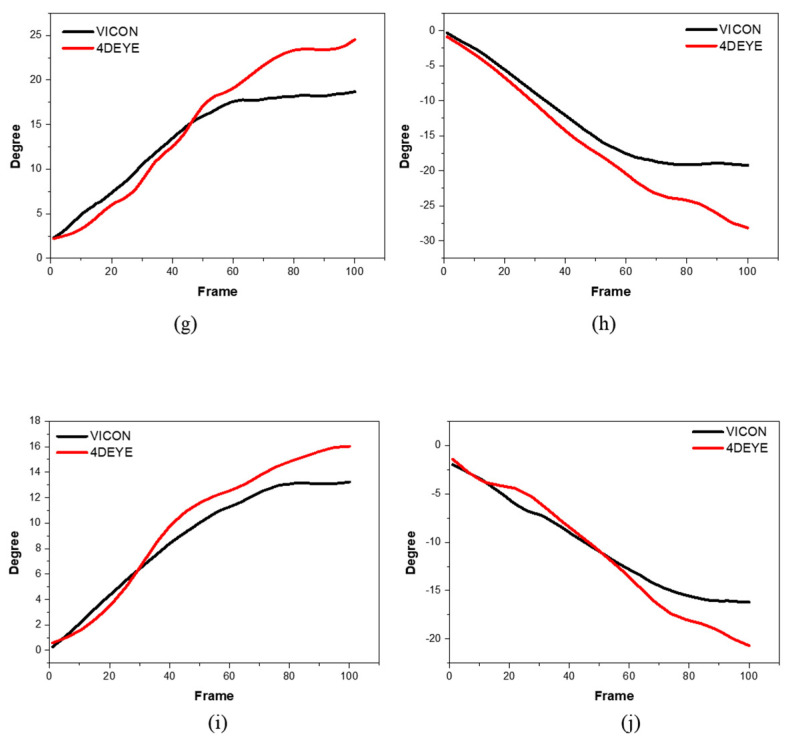
Degree of side dips using comparative analysis between VICON^®^ (black) and 4DEYE^®^ (red) with left and right measurements. (**a**) Participant 1, left; (**b**) Participant 1, right; (**c**) Participant 2, left; (**d**) Participant 2, right; (**e**) Participant 3, left; (**f**) Participant 3, right; (**g**) Participant 4, left; (**h**) Participant 4, right; (**i**) Participant 5, left; (**j**) Participant 5, right.

As presented in Figure 3, all graphs on the right (Figure 3b,d,f,h,j) show a downward trend. As this is expressed as a positive (+) when 4DEYE^®^’s trunk side bending is performed to the left and a negative (−) when performed to the right, VICON^®^ also reverses the result value to express it as a graph. 

### 3.2. Analysis of the YBT

**Figure 4:**
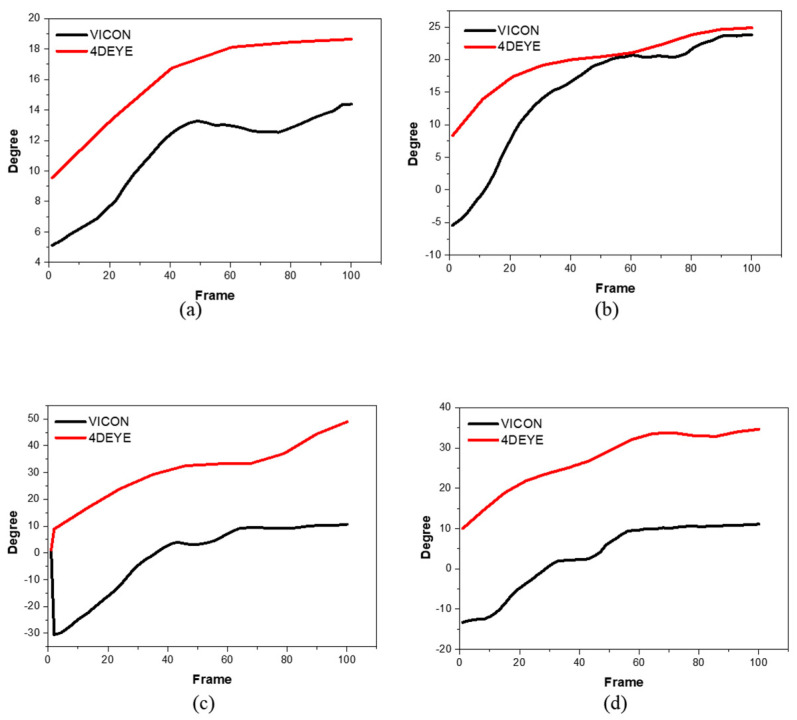

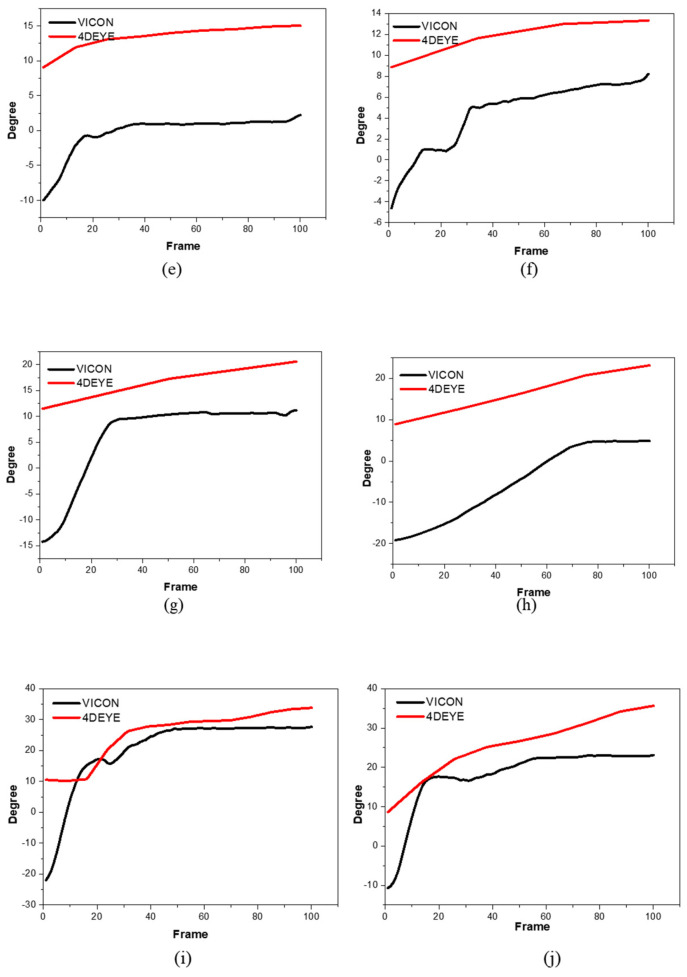
Degree of Y-balances using comparative analysis between VICON^®^ (black) and 4DEYE^®^ (red) with left and right measurements. (**a**) Participant 1, left; (**b**) Participant 1, right; (**c**) Participant 2, left; (**d**) Participant 2, right; (**e**) Participant 3, left; (**f**) Participant 3, right; (**g**) Participant 4, left; (**h**) Participant 4, right; (**i**) Participant 5, left; (**j**) Participant 5, right.

The postural movement was overlapped, the symptoms of data splashing in VICON^®^ were severely issued, and the movement in the posterior-lateral direction was selected and implemented to understand the flow of data normally. In the Y-balance graph, 4DEYE^®^ showed a higher level of data (Figure 4).

### 3.3. Statistical Analysis

The ICC result values were categorized as follows: <0.4 (poor), 0.4–0.6 (normal), 0.6–0.75 (good), and 0.75–1.00 (very good). As shown in Table 1, the ICC values for the side dips and Y-balance were generally very high (>0.75). This is judged to indicate high consistency and reliability between the two inspection methods implemented using the VICON^®^ and 4DEYE^®^ equipment. Individual data are presented in Table 1.

## 4. Discussion

In this study, we aimed to measure the movement angle of the hip extension motion formed through a combination of CKC and sagittal plane movements using 4DEYE^®^. The number of frames of 4DEYE^®^ is lower than that of VICON^®^, so low-speed simple and single-plane operations are more suitable than complex high-speed operations. And the core purpose of this study is not to look at plane or complex movements, but to check how reliable joint angle measurements are according to the classification of the Human Kinetic Chain (Open or Closed). Additionally, the investigation of motion through a 3D motion capture system utilizing three or more RGB sensors is a novel endeavor. The research was initiated with a focus on a single plane. The analysis of complex movements and fast movements will be conducted sequentially because it is necessary to analyze various complex movements related to inversion and sports.

In the analysis of the side dip, the X-axis is a frame, the maximum and minimum sections are cut, and the frames in the section are matched with 100 through VICON^®^ and 4DEYE^®^ to produce the results and graph. Subsequently, the correlation coefficient was obtained from the statistical analysis of the results. The Y-axis represents the angles of movement while performing side dip and YBT exercises. The body angle was obtained by dividing the frame of the sample motion by a constant time. In VICON^®^ and 4DEYE^®^, the degree value increases as the number of frames increases over time. This result was the same for subsequent side dip and YBT analyses.

Side dip was effective in increasing the overall core strength. The oblique muscles are a major component of the core and are often neglected. Strengthening the oblique muscles with side bends increases the strength and stability of the core. The primary muscles used for side bending are the external/internal oblique, quadratus lumborum, and erector spinae muscles. The quadratus lumborum muscles are located deep on the sides of the lower back. As shown in the following graph, VICON^®^ and 4DEYE^®^ are consistently high, with similar upward and downward tendencies on both the left and right sides. In addition, the 4DEYE^®^ equipment measured at a higher level in the right-up direction than in the VICON^®^ equipment, and at a lower level in the right-down direction. The 4DEYE^®^ has lower frequency data than the VICON^®^ equipment; therefore, the data value is wide. Moreover, 4DEYE^®^ and VICON^®^ use different methods for indicating the indicators.

In the analysis of the YBT, the YBT test was used to determine how dangerous a person is when injured. YBT can be used in upper and lower branches, which have been studied a lot since its protocol is used as a major basis for research on star travel balance tests. For example, the star travel balance test showed high reliability results in predicting lower limb injury in high school basketball players [30], and therefore, LQYBT was excellent at identifying players at increased risk of injury [31]. The upper branch YBT (UQYBT) test is a highly reliable test used to analyze unilateral upper limb (UE) function at closed chain positions. UQYBT also allows the patient to reach three directions with one UE while in the push-up position [25]. Additional re-investigation is needed to determine the application of UQYBT. In LQYBT, the patient stands on one leg and reaches forward and backward from the other lower extremities. When using the YBT kit, the points of arrival can yield “complex reach” or composite scores used to predict injury. For example, previous studies have shown that college football players with a combined score of <89% have an increased likelihood of injury from 37.7% to 68.1% [31]. Therefore, the cutoff point of 89% compound reach in YBT was determined (with sensitivity 100%, +LR 3.5) [31]. These studies revealed that each sport/population has its own risk cut point [30,31]. LQYBT showed the reliability of good intermediates or retested subjects with acceptable levels of measurement errors among multiple raters screening active service members, and the second study showed good reliability (ICC, 0.88–0.99) [29,32]. In this study, it was difficult to proceed in all three directions of Y Balance continuously.

The limitations of this study are as below. In Statistical analysis, 4DEYE^®^ is measured from ≥10° without noise and rises consistently. In VICON^®^, the movement begins at the start of the operation in the operation of hip extension in the Y-balance posterior-lateral direction while the graph breaks at the 10th-degree line, showing a shape very similar to that of 4DEYE^®^. This should reflect the mechanical characteristics of the two pieces of equipment. However, if the equipment does not increase by more than a sufficient angle when determining movement, it is not judged to have moved. Therefore, guidelines related to this are necessary. In addition, the gap between the two graphs is large because 4DEYE^®^ estimates trunk side-bending through the angle of the line connecting the left and right lines of the floor coordinates and both shoulders, whereas VICON^®^ is measured with a marker directly on the spine; therefore, there may be variations in angles between them depending on the measurement and estimation methods.

## 5. Conclusions

In this study, the reliability of a newly developed multi-view RGB sensor-based markerless motion analysis device was compared with that of an existing marker-attached motion analysis device with the highest joint measurement reliability. Regardless of performing CKC and OKC exercises, the joint measurement results were similar to those of the existing devices, confirming the high reliability of the newly developed multi-view RGB sensor. As this new multi-view RGB sensor-based motion analysis equipment does not require a marker and uses general RGB sensors, it significantly lowers the cost of motion analysis, thereby increasing convenience and economy. In terms of technology, multi-view motion images captured in various directions have been introduced to secure 3D musculoskeletal movements. In addition, a quantitative evaluation technique that does not require post-processing is possible. Therefore, it is expected to be in the spotlight by service providers (medical personnel or fitness experts) in the musculoskeletal healthcare field, and unlike conventional subjective and qualitative musculoskeletal healthcare services composed of visual measurements, it is expected to provide differentiated services based on quantitative musculoskeletal motion data with scientific evidence and standardized protocols. Furthermore, it is expected to lay the foundation for expanding to various fields based on big data and creating new service opportunities in the musculoskeletal healthcare field in the future, such as the general health examination field, by continuously using quantitative big data on musculoskeletal movements and information on expected diseases predicted from movements. In subsequent studies, movements that occur by combining multiple joints and various movements in a continuous sequence should be selected and analyzed for joint measurement values to determine whether a more in-depth motion analysis is possible.

## Figures and Tables

**Table 1:** Statistical analysis of 4DEYE^®^ related to VICON^®^ measurements.

Movement		Left				Right			
Body Regions	Participants	M	SD	ICC(2.1)	95% CI	M	SD	ICC(2.1)	95% CI
Side dip (Trunk side-bending)	1	14.48	11.27	0.895	0.843–0.929	−29.61	10.40	0.985	0.978–0.990
	2	43.50	18.13	0.969	0.954–0.979	−39.30	20.30	0.978	0.968–0.985
	3	34.30	11.67	0.996	0.993–0.997	−23.70	8.78	0.938	0.909–0.959
	4	28.45	12.93	0.966	0.950–0.977	−29.18	14.76	0.974	0.961–0.982
	5	18.72	9.28	0.966	0.979–0.990	−21.76	10.78	0.974	0.962–0.983
Y-balance (Hip extension)	1	27.54	5.30	0.973	0.960–0.982	35.21	12.64	0.859	0.791–0.905
	2	30.23	22.58	0.949	0.924–0.966	30.68	15.12	0.990	0.984–0.993
	3	13.33	4.11	0.879	0.821–0.919	16.46	4.45	0.833	0.752–0.888
	4	23.04	9.95	0.678	0.521–0.783	11.37	13.07	0.887	0.832–0.924
	5	45.96	18.98	0.886	0.831–0.923	43.76	14.52	0.924	0.887–0.949

CI, confidence interval; ICC, intraclass correlation coefficient.

## Data Availability

No new data were created or analyzed in this study. Data sharing is not applicable to this article.
